# Treatment of perianal abscess and anal fistula in infants: a systematic review

**DOI:** 10.3389/fsurg.2025.1572049

**Published:** 2025-06-13

**Authors:** Jinlan Chen, Yi Xiong, Cong Wang, Li Xu

**Affiliations:** First Clinical Medical College of Zhejiang Chinese Medical University, Hangzhou, Zhejiang, China

**Keywords:** infants, perianal abscess, fistula-in-ano, conservative treatment, surgical treatment

## Abstract

**Introduction:**

The optimal treatment approach for perianal abscess (PA) and fistula-in-ano (FIA) in infants remains a subject of debate.

**Material and Methods:**

A thorough literature search was conducted across multiple databases, including Embase, PubMed, Web of Science, Cochrane Library, ClinicalTrials.gov, and Google Scholar.

**Results:**

A total of eighteen retrospective studies, encompassing 1,770 patients, were analyzed. Of the 702 cases (38.7%) that underwent conservative treatment, 528 cases (75.2%) achieved a cure, while 174 cases (24.8%) were unsuccessful, with 93 experiencing PA recurrence and 81 developing FIA. In contrast, among the 1,068 cases (61.3%) that received surgical interventions, 784 cases (73.4%) were cured, whereas 284 cases (26.6%) were not, with 151 experiencing PA recurrence and 133 developing FIA.

**Conclusion:**

The available studies indicate minimal differences in the cure and recurrence rates of PA and FIA between the conservative and surgical treatment groups.

**Systematic Review Registration:**

https://www.crd.york.ac.uk/PROSPERO/, PROSPERO (CRD42023472249).

## 1 Introduction

Perianal abscess (PA) is a purulent condition characterized by either acute or chronic infection of the tissues surrounding the anorectal region, primarily resulting from infection of the anal gland. Clinical manifestations of PA include erythema and pain in the perianal area, often accompanied by induration, with or without the sensation of fluctuation, and typically measuring between 0.5 and 3.0 cm in diameter. Rupture of the abscess may lead to the formation of a pathological tract connecting the anorectum to the external environment, referred to as a fistula-in-ano (FIA) (1). This condition can progress from an acute perianal abscess to a chronic anal fistula (2). This progression underscores the clinical importance of timely and effective management to prevent long-term complications.

From an epidemiological perspective, perianal abscess (PA) and fistula-in-ano (FIA) exhibit significant susceptibility within the pediatric population, with over 75% of affected infants presenting initial symptoms before reaching one year of age (3). Studies consistently report a notable male predominance, although the underlying causes remain unclear. While the precise pathogenesis of PA has yet to be fully elucidated, current hypotheses suggest congenital anomalies of the anal glands, hormonal influences—particularly androgenic effects on glandular activity in male infants—and local trauma or micro-tears that facilitate bacterial invasion (4). It is noteworthy that the natural progression of infantile PA differs markedly from that observed in adults. In adults, the pathogenesis predominantly aligns with the cryptoglandular infection theory, wherein obstruction of anal gland ducts leads to bacterial proliferation and abscess formation, with subsequent rupture potentially developing into chronic fistulae (5). Importantly, the presence of complex or recurrent fistulae should prompt suspicion for Crohn's disease (CD), as 30%–50% of CD patients develop concurrent PA or FIA, typically presenting as multiple, complex fistulae with recurrence rates reaching 60% (6). Adult patients exhibit a slight male predominance and generally require surgical intervention, with a strong association with CD, particularly in younger patients who warrant screening (7). In contrast, infantile cases display a pronounced male predominance, likely due to congenital developmental anomalies, and primarily present as superficial simple fistulae with a rare association with CD. These fundamental differences significantly influence clinical decision-making: adult patients necessitate proactive surgical management with the exclusion of CD, while cases associated with CD require a combination of biologic and surgical therapy. Pediatric cases may resolve spontaneously; however, some may progress to chronic fistulation or recurrent abscess formation, necessitating clinical intervention.

Therapeutic strategies for infantile PA and FIA primarily involve conservative and surgical approaches. For uncomplicated or early-stage cases, conservative management typically includes oral or topical antibiotics, anti-inflammatory ointments, and traditional Chinese medicine sitz baths to reduce inflammation and promote drainage.

In adult patients, surgical interventions, such as incision and drainage, fistulotomy, or seton placement, continue to be the gold standard for definitive treatment due to the high likelihood of fistula formation following abscess rupture (8). In contrast, treatment strategies for infants remain contentious. Some clinicians advocate for conservative management, citing the potential for spontaneous resolution and the risks associated with surgery, while others emphasize early surgical intervention to prevent recurrence and fistula development.

Despite ongoing debate within the academic community regarding the optimal treatment strategies for PA and FIA in infants, the current evidence is significantly inadequate to establish reliable clinical guidelines. Specifically, existing studies face three critical limitations: first, many studies have limited sample sizes that do not achieve sufficient statistical power; second, there is considerable heterogeneity in research methodologies, complicating direct comparisons of results; and third, the lack of long-term follow-up data hinders the evaluation of the long-term efficacy of different treatment approaches. More importantly, no internationally recognized clinical guidelines have been established for this special patient population. The absence of standardized treatment protocols leaves clinicians without unified criteria when formulating therapeutic plans. It is precisely to address this pressing clinical dilemma that our study aims to systematically evaluate the existing evidence, thereby providing an evidence-based foundation for standardizing the treatment of PA and FIA in infants.

A significant gap exists in the field due to the absence of internationally recognized clinical guidelines tailored to this specific patient population. This lack of standardized treatment protocols presents a challenge for clinicians in developing consistent and evidence-based therapeutic strategies. To address this critical clinical need, our study aims to systematically evaluate the available evidence, with the goal of establishing an evidence-based framework for standardized treatment of PA and FIA in infants.

## 2 Material and methods

### 2.1 Search strategy

Two independent reviewers conducted a comprehensive search of six major databases (Embase, PubMed, Web of Science, Cochrane Library, ClinicalTrials, and Google Scholar) for articles on the conservative or surgical treatment of perianal abscess (PA) and fistula-in-ano (FIA) in infants, covering the period from January 1990 to November 2023. The search utilized key MeSH terms, including “infant,” “newborn,” “baby,” “perianal abscess,” “fistula-in-ano,” and “anal fistula,” along with all relevant combinations. Additionally, the reference lists of included studies were manually searched to identify any additional relevant articles. We restricted the search to studies published in English, involving human subjects, and focused on infants and toddlers under the two years of age. Studies were excluded if they were cadaveric, non-human, case reports, technical notes, or qualitative studies. This review adhered to the PRISMA and AMSTAR 2 guidelines (9, 10), and was registered in PROSPERO (CRD42023472249).

### 2.2 Types of interventions

We included articles published in English that focused on the conservative or surgical treatment of perianal abscess (PA) and fistula-in-ano (FIA) in infants. The primary outcome measures were the recurrence rates of PA and the incidence of FIA formation following conservative or surgical interventions.

### 2.3 Study screening

Two reviewers independently evaluated the titles, abstracts, and full texts of the articles. Any discrepancies were discussed and resolved through collaborative deliberation. The references of the included studies were also reviewed to identify any additional relevant articles.

### 2.4 Data abstraction

Data extraction from the selected studies was conducted independently by two reviewers. The extracted data were documented using Excel 2019. The characteristics of the studies extracted included the author, year of publication, country of study, sample size, proportion of male participants, mean age, and follow-up duration. Additionally, clinical data and descriptive statistical data were collected. In cases where data distribution was unavailable, the means of the ranges were utilized.

## 3 Statistical analysis

This study was meticulously conducted in alignment with the PRISMA 2020 statement guidelines for systematic reviews. Due to the considerable variability in conservative treatment approaches across the included studies—encompassing differences in antibiotic regimens, treatment durations, oral medications, and local management protocols—as well as the pronounced statistical and methodological heterogeneity observed, a meta-analysis was deemed inappropriate. Consequently, the findings were synthesized through descriptive methods. Descriptive statistics were systematically presented within the text, encompassing measures such as means and mean differences, with all variables reported as mean ± standard deviation (SD). The primary efficacy outcomes assessed were the cure rate and recurrence rate. The cure rate was determined using the formula (number of cured cases/total cases in the group) × 100%, while the recurrence rate was calculated as (number of recurrent cases/total cases in the group) × 100%.

## 4 Results

### 4.1 Study characteristics

**Figure 1:**
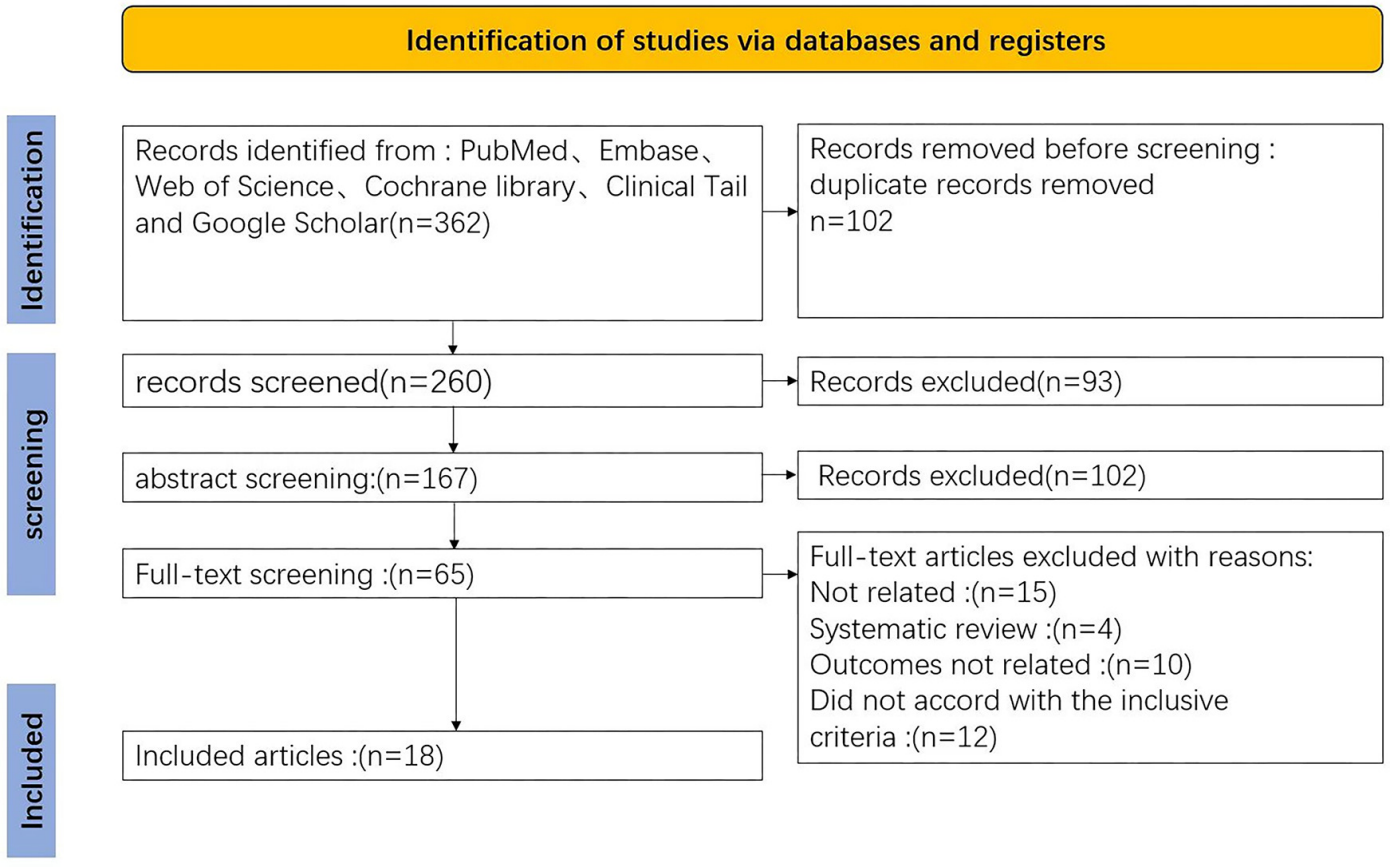
The PRISMA flowchart.

The initial search identified 362 articles. Following the removal of 102 duplicates and a thorough screening of titles, abstracts, and full texts, 18 articles were ultimately included in the analysis. All selected studies were retrospective in nature. The PRISMA flowchart is presented in Figure 1.

**Figure 2:**
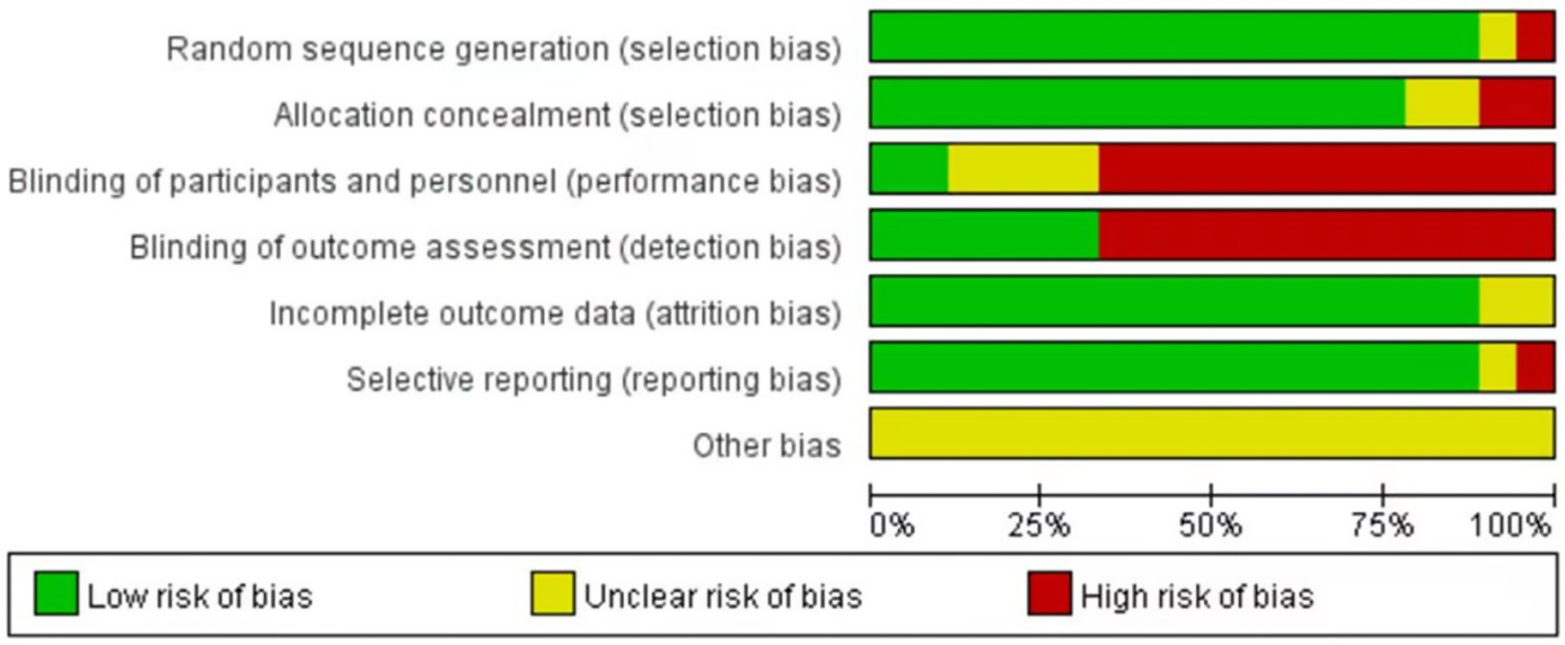
Bias risk map.

These 18 studies, published over the past 33 years (1990–2023), satisfied the inclusion criteria. Each included study was subjected to an impartial quality assessment, with the risk of bias illustrated in Figure 2. Collectively, these studies encompassed a total of 1,770 infants diagnosed with PA or FIA, the majority of whom were male (1,499; 84.7%). The median age of the patients was 6.5 months (2.2–24), and none of the infants or toddlers had underlying medical conditions. The characteristics of the included studies are summarized in Table 1.

**Table 1:** Summary of the included studies.

Study	Duration	Country	Total number	Mean age(months)	Males	Follow up(months)
Erten et al. (22)	January 2014-January 2022.	Turkey	251	24	230 (92%)	19.3
Kang et al. (18)	January 2010 to December 2020	China	477	2.2	378 (79.2%)	12
Hanada et al. (19)	April 2011-March 2014	Japan	45	4.3	45 (100%)	26
Emily et al. (20)	January 1995-February 2005	Canada	140	4.2	132 (94%)	NA
Serour et al. (31)	January 1990-June 2002	Israel	77	6.9	75 (97%)	3.2
Afsalar et al. (21)	January 2005-December 2010	Turkey	136	7.2	136 (100%)	10.6
Balaz et al. (23)	January 2013-December 2015	Poland	24	4	24 (100%)	24
Chang et al. (16)	January 2006-December 2008	South Korea	100	6	100 (100%)	26
Festen et al. (28)	January 1974-December 1994	Netherlands	26	5	26 (100%)	92.4
Wanbin et al. (15)	September 2014-December 2020	China	153	4	149 (97.4%)	63.6
Sharman et al. (26)	2007–2017	Australia	111	2.1	110 (99.1%)	NA
Samuk et al. (17)	August 2014-January 2018	Israel	19	8.4	19 (100%)	22.4
David et al. (25)	July 1978-May 1989	America	22	9	22 (100%)	NA
Poenaru et al. (24)	1980–1991	Canada.	31	9.5	31 (100%）	20
Rosen et al. (14)	1990–1999	America	18	6.7	18 (100%)	37
Ikeda et al. (30)	May 2003-April 2005	America	14	4.6	14 (100%)	1.2
Inoue et al. (29)	January 2006-February 2010	Japan	90	3.1	90 (100%)	49.8
Gafar et al. (27)	January 2010-December 2011	Egypt	36	9.7	36(100%)	1.2

### 4.2 Treatment of PA and FIA

#### 4.2.1 Conservative treatment of Pa and FIA

In a review of 10 studies, 702 infants, accounting for 38.7% of the total, received conservative management. This approach primarily encompassed sitz baths, antibiotics, oral Chinese medicine (TJ-122), combinations of antibiotics and sitz baths, sitz baths with topical ointment, and spontaneous drainage, as detailed in Table 2.

**Table 2:** Conservative treatment methods in the included studies.

Study	Sitz bath	Antibiotics	TJ-122	Antibiotics and sitz bath	Sitz bath and topical ointment
Kang et al.	0	0	0	189	0
Handa et al.	0	0	19	0	0
Emily et al.	0	57	0	0	0
Serour et al.	25	0	0	0	0
Afsalar et al.	0	36	0	0	0
Chang et al.	0	100	0	0	0
Samuk et al.	0	19	0	0	0
Wanbin et al.	0	0	0	0	153
Rosen et al.	0	18	0	0	0
Total number	25	230	19	189	153

#### 4.2.2 Surgical treatment of PA and FIA infants

In 14 studies, 1,068 infants, representing 61.3% of the total, underwent surgical intervention. Specifically, 845 infants underwent incision and drainage, 85 underwent incision and drainage with fistulotomy, 47 underwent needle aspiration, 35 underwent fistulotomy, 32 underwent fistulectomy, and 24 underwent seton placement, as presented in Table 2.

### 4.3 Outcomes after conservative and surgical treatment

#### 4.3.1 Cure and failure rates after conservative treatment

Of the 702 cases (38.7%) that underwent conservative treatment. In the cohort receiving conservative treatments, 25 patients underwent sitz bath therapy, 230 were administered antibiotics, 19 received oral TJ-122, 189 were treated with a combination of antibiotics and sitz bath therapy, and 153 were managed with sitz bath therapy in conjunction with topical ointment application. Of these, 528 patients (75.2%) achieved successful outcomes, whereas 174 patients (24.8%) experienced treatment failure. Among the failed cases, PA recurred in 93 instances, and FIA developed in 81 instances, as detailed in Table 2.

#### 4.3.2 Cure and failure rates after surgical treatment

A total of 1,068 cases (61.3%) received surgical intervention. In the cohort undergoing surgical interventions, 86 patients underwent spontaneous drainage, 845 received incision drainage, 85 were treated with incision drainage combined with fistulotomy, 47 underwent needle aspiration, 35 had fistulotomy alone, 32 underwent fistulectomy, and 24 were treated with seton placement. Among these patients, 784 (73.4%) were successfully cured, while 284 (26.6%) experienced treatment failure. Within this group of failed cases, PA recurred in 151 instances, and FIA developed in 133 instances, as illustrated in Tables 3, 4.

**Table 3:** Surgical management methods in the included studies.

Study	Spontaneous drainage	Incision drainage	Incision drainage and Fistulotomy	Needle aspiration	Fistulotomy	Fistulectomy	Seton
Erten et al.	86	165	0	0	0	0	0
Kang et al.	0	288	0	0	0	0	0
Handa et al.	0	26	0	0	0	0	0
Emily et al.	0	83	0	0	0	0	0
Serour et al.	0	5	0	47	0	0	0
Afsalar et al.	0	100	0	0	0	0	0
Balaz et al.	0	24	0	0	0	0	0
Festen et al.	0	26	0	0	0	0	0
Sharman et al.	0	26	85	0	0	0	0
David et al.	0	22	0	0	0	0	0
Poenaru et al.	0	0	0	0	17	14	0
Ikeda et al.	0	0	0	0	0	0	14
Inoue et al.	0	80	0	0	0	0	10
Gafar et al.	0	0	0	0	18	18	0
Total number	86	845	85	47	35	32	24

**Table 4:** Summary of outcomes of conservative management and surgical management.

Study	Cure of conservative treatment (number/tot)	Failure of conservative treatment (number/total)	Recurrence of PA after conservative treatment	Formation. of FIA after conservative treatment	Cure of surgery (number/total)	Failure of surgery (number/total)	Recurrence of PA after surgical treatment	Formation. of FIA after surgical treatment
Erten et al.	62/86	24/86	14/86	10/86	162/165	3/165	3/165	0/165
Kang et al.	127/189	62/189	22/189	40/189	203/288	85/288	35/288	50/288
Handa et al.	18/19	1/19	1/19	0/19	24/26	2/26	2/26	0/26
Emily et al.	48/57	9/57	6/57	3/57	33/83	50/83	27/83	23/83
Serour et al.	15/25	10/25	7/25	3/25	32/52	20/52	11/52	9/52
Afsalar et al.	15/36	21/36	12/36	9/36	53/100	47/100	27/100	20/100
Chang et al.	97/100	3/100	3/100	0/100	0	0	0	0
Wanbin et al.	119/153	34/153	20/153	14/153	0	0	0	0
Samak et al.	13/19	6/19	4/19	2/19	0	0	0	0
Rosen et al.	14/18	4/18	4/18	0/18	0	0	0	0
Balaz et al.	0	0	0	0	23/24	1/24	0/24	1/24
Festen et al.	0	0	0	0	17/26	9/26	6/26	3/26
Sharman et al.	0	0	0	0	89/111	22/111	12/111	10/111
David et al.	0	0	0	0	12/22	10/22	6/22	4/22
Poenaru et al.	0	0	0	0	27/31	4/31	4/31	0/31
Ikeda et al.	0	0	0	0	13/14	1/14	0/14	1/14
Inoue et al.	0	0	0	0	63/90	27/90	17/90	10/90
Gafar et al.	0	0	0	0	33/36	3/36	1/36	2/36
Total number	528/702	174/702	93/702	81/702	784/1,068	284/1,068	151/1,068	133/1,068

#### 4.3.3 The cure rate, failure rate, PA recurrence rate and FIA formation rate after conservative and surgical treatment

The cure rate associated with conservative treatment was 75.2%, while the failure rate stood at 24.8%. The recurrence rate for PA was observed to be 13.2%, and the rate of FIA formation was 11.5%. In contrast, surgical treatment exhibited a cure rate of 73.4% and a failure rate of 26.6%. The PA recurrence rate for surgical treatment was 14.1%, with an FIA formation rate of 12.5%. Comprehensive results are presented in Table 5.

**Table 5:** Cure, failure, PA recurrence and FIA formation rates after conservative and surgical treatment.

Outcome	Cure rate	Failure rate	Recurrence rate of PA	Formation rate of FIA
Conservative treatment	75.2%	24.8%	13.2%	11.5%
Surgical treatment	73.4%	26.6%	14.1%	12.5%

## 5 Discussion

Currently, there is a lack of consensus regarding the most effective treatment strategy for PA and FIA in infants. Both conservative and surgical approaches offer unique benefits and drawbacks. Surgical intervention, which entails incision and drainage of the PA as well as resection of the FIA, effectively minimizes the risk of recurrence, expedites symptom relief, prevents the dissemination of infection, and reduces the duration of medical management. Nevertheless, surgical treatment is associated with several potential disadvantages, including the risks related to anesthesia, bleeding, surgical complications, and postoperative infection. In contrast, conservative treatment eliminates the risks associated with anesthesia, surgical incision wounds, and the need for hospitalization. However, it presents several drawbacks, such as a high likelihood of recurrence and an extended treatment duration. This article evaluates existing literature to determine the most effective treatment modality for PA and FIA in infants.

The pathogenesis of perianal abscess (PA) and fistula-in-ano (FIA) exhibits distinct characteristics across various age groups. In adults, these conditions predominantly arise from bacterial infections, primarily involving Escherichia coli and anaerobes, affecting the perianal glands and leading to suppurative inflammation. Contributing factors include local trauma and hygiene conditions (5). Conversely, current evidence in infants supports a multifactorial developmental hypothesis, which includes elevated androgen levels (particularly in male infants) stimulating excessive anal gland secretion, immature immune system development (such as deficient secretory immunoglobulin A production), abnormal gut microbiota colonization, and local microtrauma resulting from improper care (11). Notably, there is a significant association between adult PA/FIA and Crohn's disease (CD), with approximately 30%–50% of CD patients developing perianal lesions, primarily mediated by transmural intestinal inflammation penetrating the bowel wall (12, 13). In contrast, the correlation between infantile PA/FIA and CD is considerably weaker. This disparity indicates that adult presentations of PA and FIA, particularly those that are complex and recurrent, should be screened for CD, whereas cases in infants likely reflect distinct developmental pathological processes. Understanding these age-related pathogenic differences is essential for the development of tailored treatment strategies.

A systematic review of 1,770 infant cases revealed that the recurrence rates for both conservative and surgical treatments were similar, prompting a reevaluation of the optimal treatment strategy. Current research offers two primary perspectives: Scholars such as Rosen (14), Wanbin (15), Chang (16), and Samuk (17) highlight the self-limiting nature of infant PA and FIA, advocating for conservative treatment as the preferred approach. Kang et al. (18) recommend avoiding immediate surgical intervention for most initial cases of PA and FIA. Hanada et al. (19) propose that oral TJ-122 treatment for infant PA is more advantageous than incision and drainage. Emily et al. (20). suggest that the development of perianal abscesses (PA) in infants under 12 months differs from that in older infants and propose that local sitz bath therapy combined with oral antibiotics can reduce the formation of anal fistulas. Conversely, Afsarlar et al. (21) argue that PA drainage coupled with antibiotic treatment decreases the incidence of anal fistulas, and that infant fistula-in-ano (FIA) may resolve spontaneously, thereby rendering immediate surgical intervention unnecessary.

In contrast, researchers such as Erten (22), Bałaż (23), Poenaru (24), David (25), and Sharman (26) advocate for proactive surgical management, asserting that incision and drainage with antibiotics, or fistulotomy and fistulectomy, can significantly lower recurrence rates while providing improved long-term outcomes and enhanced patient satisfaction. Furthermore, there is ongoing debate concerning the optimal surgical approach. Gafar et al. (27) report that fistulotomy is superior to fistulectomy in terms of recovery time and complication rates, whereas Festen et al. (28) highlight the superior long-term cure rate associated with fistulectomy.

Inoue (29) and Ikeda (30), along with their colleagues, propose that seton treatment should be implemented for infantile FIA, as the majority of patients experience recovery following the first or second seton placement, with an average recovery period of approximately one and a half months. Based on the current evidence, we are inclined to endorse the stepwise treatment approach advocated by Serour et al. (31), which involves conservative management through local treatment and antibiotics for a duration of 1–3 months in early-stage cases, reserving surgical intervention for persistent fistulas. This personalized treatment strategy considers the distinct pathophysiological characteristics of infants, such as androgen levels and immune system development, while minimizing the trauma associated with excessive surgical procedures. This approach is consistent with most research findings. Nevertheless, it is crucial to emphasize that clinical decisions should be grounded in a comprehensive assessment of each child's specific condition. Future research should prioritize rigorously designed prospective studies to validate the long-term efficacy of various treatment modalities, with a particular focus on establishing standardized outcome assessment criteria and uniform surgical protocols.

This study is subject to several limitations: there was notable heterogeneity in the diagnostic criteria and outcome measures among the included studies; potential selection bias may have influenced treatment allocation; most studies lacked long-term follow-up data, complicating the assessment of long-term outcomes; furthermore, the reporting standards for comorbidities were inconsistent across studies; and finally, conservative treatment protocols were not standardized. These factors may collectively impact the interpretation and generalizability of the study findings.

This study has several limitations: there was significant heterogeneity in the diagnostic criteria and outcome measures across included studies; potential selection bias may exist in treatment allocation; most studies lacked long-term follow-up data, making it difficult to assess long-term outcomes; additionally, reporting standards for comorbidities were inconsistent among studies; finally, conservative treatment protocols lacked standardized approaches. These factors may collectively influence the interpretation and generalizability of the study findings.

Based on the findings of this study, it is recommended that the management of perianal abscesses in infants be guided by the underlying pathogenetic condition and the preferences of the parents. Conservative management is advised for infants presenting in the early stages of abscess formation, exhibiting mild symptoms, or for those who are unsuitable candidates for surgical intervention. This strategy may decrease the likelihood of surgical incision and associated complications. Conversely, for infants who exhibit resistance to conservative management, experience recurrent symptoms, or present with severe systemic manifestations, surgical intervention becomes imperative. The selection of the surgical technique is contingent upon the pathogenetic condition of the infant. During the procedure, it is imperative to achieve complete resection of the infected tissues while preserving anal function. Postoperative management should focus on preventing constipation and diarrhea, ensuring meticulous wound care, and mitigating the risk of complications. In conclusion, the appropriate therapeutic approach for varying types and severities of infant PA and FIA should be determined based on the infant's clinical presentation and the preferences of the parents.

## 5 Conclusion

The incidence of PA and FIA is significantly higher in male infants, with these conditions often resolving spontaneously as immune function matures. Our systematic review of the existing evidence indicates minimal differences in cure and recurrence rates between conservative and surgical treatments. However, it is important to acknowledge that current conclusions are predominantly derived from retrospective studies with small sample sizes and short follow-up durations, highlighting the urgent need for more high-quality studies to establish optimal treatment protocols.

## Data Availability

The original contributions presented in the study are included in the article/Supplementary Material, further inquiries can be directed to the corresponding author.
